# Telehealth and Beyond: Promoting the Mental Well-Being of Children and Adolescents During COVID

**DOI:** 10.3389/fped.2022.793167

**Published:** 2022-02-14

**Authors:** April Joy Damian, Katy Stinchfield, R. Timothy Kearney

**Affiliations:** ^1^Weitzman Institute, Community Health Center, Inc., Middletown, CT, United States; ^2^Johns Hopkins Bloomberg School of Public Health, Baltimore, MD, United States; ^3^School Based Health Alliance, Washington, DC, United States

**Keywords:** telehealth, mental healthcare access, children and adolescents, pediatrics, health equity and behavioral health

## Introduction

There is growing recognition of converging pandemics with a mental health crisis emerging alongside COVID-19. While the literature has primarily focused on the biopsychosocial impact of the pandemic on higher-risk populations (e.g., the elderly) (1–5), recent studies suggest that COVID-19 is negatively affecting the mental health of children and adolescents (6–8). Studies have shown that youth have exhibited increased irritability, inattention, disturbed sleep, and other depressive and anxious symptoms (6, 9–11), highlighting the profound impact of the pandemic on the emotional and social development on this population. Moreover, during the current pandemic, there has been greater attention and investment in telehealth, including telemental health, as a means of reducing the risk of infection among patients and healthcare workers, while still providing needed care. Telemental health includes a wide range of services including psychiatric evaluations, therapy (individual, family, and group), patient education, and medication management. Prior to the pandemic, there had already been a strong body of evidence for telehealth-based interventions. For example, the American Psychiatric Association have noted multiple studies supporting its effectiveness being comparable to in-person treatment in terms of therapeutic engagement, quality of care, validity/reliability of assessment, and clinical outcomes (12). Moreover, studies suggest that there are a few populations for whom telemental health may be preferable to in-person care, including patients with autism spectrum disorders, anxiety disorders, and/or physical limitations (13).

As senior leaders, behavioral health providers, and researchers at the School-Based Health Alliance (SBHA), the national voice for school-based health centers (SBHCs) in the United States (U.S.), and Community Health Center, Inc./Weitzman Institute, one of the largest FQHC (Federally Qualified Health Center) sponsors of SBHCs in the U.S., we recognize that it is particularly important to call to attention the impact of the pandemic on the mental well-being of low-income youth and youth of color as COVID-19 has both exposed and exacerbated the health and social inequities that these populations face. We offer our perspectives on the advantages and disadvantages of telehealth in supporting the mental well-being of marginalized youth during the pandemic, and provide recommendations for how our field can promote pediatric mental health beyond telehealth.

## Advantages of Telemental Health

### Viewpoints of Patients and Families

One of the major advantages of telehealth expressed by our pediatric patient populations and their families is the safe, ease of access to mental health services. Clients have expressed fears of going outside of their homes given the risk of COVID-19 exposure and engagement with persons who are not abiding by general safety precautions, including wearing masks and physical distancing. Telemental health (accessing mental health services via telehealth) allows them to receive comprehensive care while still abiding by stay-at-home recommendations.

Telemental health also helps patients and their families overcome structural and financial barriers to treatment. Telehealth has ensured timely mental health access to traditionally hard-to-reach groups, such as multigenerational households where parents care for elderly parents or households with young children and no childcare. Transportation has often been cited as a major barrier to health care access, with studies suggesting that transportation serves as a barrier to care for as much as 67% of the population sampled (14). Similarly, for parents who are hourly workers, the challenges of having to take time off of work, as well as the challenges of reliable transportation access and financial burdens demanded by in-person services are lifted by telemental health.

Another advantage of telehealth is its provision of a space for social connection. School-based clinicians expressed that while schools are closed, pediatric patients find therapy groups very helpful to maintain social connections and work out common issues with peers they are unable to see due to lockdown status. Through our own direct patient engagements and those of our clinical colleagues, we have found pediatric clients themselves requested that this treatment modality resume as soon as remote services were up and running. Moreover, the virtual platform affords youth and adolescents the opportunity to access professional and peer support in such a way that avoids the stigma that can be associated with visiting a mental health clinic in person.

### Viewpoints of Mental Health Providers

From the provider's perspective, one of the major advantages of telemental health is professional and personal flexibility. Providers have expressed being able to see more clients with the implementation of telehealth. For example, factors including inclement weather, particularly during winter months, and lack of access to reliable transportation have been noted as patient barriers to accessing in-person care and subsequently, contributed to no-shows. Telehealth has helped overcome these barriers. In addition, providers noted being able to see clients at a more therapeutically appropriate cadence as a result of weather and transportation challenges no longer serving as barriers to care. For example, whereas with in-person care, a provider may schedule a follow-up appointment after 1 month, with a telehealth appointment, a provider may decide to follow-up after a couple of hours or a day to monitor changes in symptoms. Similarly, telehealth affords the opportunity to conduct sessions at a time that is convenient to both clients and providers as opposed to being limited to the operating hours of a brick and mortar practice site. More specifically, telehealth promotes continuum of care, particularly in terms of allowing youth and adolescents to remotely access school-based mental health services year-round, wherein there may have previously existed a gap in care during summer months as in-person health services were subject to the physical operating hours of the school.

Another advantage from the provider's perspective is the heightened exposure to issues of family dynamics/home context, which is of particular importance given the centrality and significance of the household on a child's development. For example, a child who has expressed how frustrating a sibling's behavior can be and experiences this frustration in real time as the sibling intrudes into the session now has the opportunity to tell the therapist, “See, this is what I have been telling you about.” They also have the opportunity to practice skills immediately and garner feedback. Similarly, telehealth allows a provider to see a child in their own environment and assess for child abuse, hear screams or other informative noises in background, observe interactions with siblings and family members, and virtually meet pets and/or see the child's room or treasured possessions, bridging the traditional gap between clinical practice location and the child's real world setting. This is particularly important given the increased risk of domestic violence due to the pandemic and lockdown (15–17).

## Disadvantages of Telemental Health

Nonetheless, in spite of the significant advantages of telehealth in promoting the mental well-being of children and adolescents, we recognize the limitations and challenges of this intervention for both youth and families, as well as providers. For example, lack of privacy poses a major challenge to pediatric patients who may not have the freedom to talk openly with their mental health providers due to thin walls or someone else being in the room. Additionally, the digital divide persists for many as some children and adolescents do not have access to the technology platforms necessary to utilize virtual visits, even via phone. From the providers' perspective, the quality of engagement with pediatric patients has often times been compromised as patients may be distracted by their home environment, or technological glitches can either interrupt the session or cut it short completely, thereby interrupting the flow of sessions. Similarly, the challenge of screen fatigue as young patients and provider alike are now reliant on video conferencing to conduct their respective work, has also been expressed as a disadvantage. Lastly, while the increased scheduling flexibility has its advantages in terms of supporting patient-provider engagement, the enhance flexibility and access for pediatric patients may also inadvertently be a contributor to provider burnout.

## Discussion

Though our clinical and administrative efforts at the national, state, and local levels have made it clear that telehealth plays a pivotal role in healthcare delivery, particularly during the current public health crisis, we also recognize the limitations of this innovation. Thus, while we support the ongoing use and expansion of telehealth, we also recognize the need for our field to explore and implement other mechanisms for promoting mental health of children and adolescents, which we outline here.

### Strengthening the Leadership of SBHCs and Their Education Partners at Both the National and Local Levels

As noted in our recommendations to the Biden-Harris Presidential Transition Team (18), there is a need to support the leadership of SBHCs at different levels of influence. More specifically, at the local level, we recommend that there be adequate funding allocated to ensure that Title I schools, Community Schools, and other schools serving low-income households have an SBHC. Additionally, we believe that specific relief programs and federal education funds should be allocated and set aside for school district superintendents and principals to use to their discretion to successfully and adequately re-open, re-stock, and re-start schools and SBHCs when the pandemic ends. At the national level, we recommend creating a White House Council and Office on Children and Youth and giving SBHA a seat on that Council. Moreover, we recommend that the office be staffed with people who have demonstrated experience and commitment to serving the whole child and recognize the critical role that healthcare plays in that process.

### Building the Competency and Resiliency of the Existing Youth-Serving Workforce

Providing and expanding pediatric health and mental health services is strongly dependent on an adequately trained and supported workforce. Thus, we concur with the National Center for School Mental Health's recommendations to invest in the professional development of youth-facing staff, particularly in the areas of adversity, trauma, toxic stress, resilience, and cultural humility and responsiveness. For example, healthcare personnel placed in schools should be well positioned to train teachers, school administrators, and other paraprofessionals on universal screening and monitoring for mental health concerns, including assessments of other social determinants of health (19). Similarly, there is also a need to ensure that adequate support systems are in place to address secondary traumatic stress that may arise among youth-facing professionals. Preventing secondary traumatic stress among youth-facing personnel includes processes such as assessing the mental health needs of employees, normalizing help-seeking behavior to reduce stigma, and compensating staff time to access needed support. Additionally, local resources for peer support could be shared with staff as a means of increasing awareness of peer support unique to the local community while further destigmatizing help-seeking behaviors (20).

### Expanding and Diversifying the Mental Health Workforce

We know that youth mental health is suffering due to COVID-19, and that youth of color and those living in low-income households are disproportionately affected (21). Moreover, there is a strong body of literature supporting the need to increase the proportion of racial/ethnic minority providers as a means for promoting culturally and linguistically competent care (22). Thus, dedicating Federal funds toward expanded recruitment and retention of a diverse mental healthcare workforce, and creating incentives such as tax credits and student loan forgiveness programs to attract highly qualified staff to work in schools and communities with identified mental health service shortages, are key policy recommendations for addressing the mental health needs of vulnerable youth as well as reducing health disparities.

We recognize that pediatric mental health has been sidelined during the current public health crisis, and reiterate the urgency required to address this double pandemic. To move forward, we must invest in targeted, collaborative personnel and community structures that can best deliver essential services to our most vulnerable young people, where they are most accessible: in school. Children and adolescents deserve no less.

## Author Contributions

AJD and RTK: study conception and design, analysis, and interpretation of the results. AJD and KS: data collection review of the literature. AJD: draft manuscript preparation. All authors reviewed the results and approved the final version of the manuscript.

## Conflict of Interest

The authors declare that the research was conducted in the absence of any commercial or financial relationships that could be construed as a potential conflict of interest.

## Publisher's Note

All claims expressed in this article are solely those of the authors and do not necessarily represent those of their affiliated organizations, or those of the publisher, the editors and the reviewers. Any product that may be evaluated in this article, or claim that may be made by its manufacturer, is not guaranteed or endorsed by the publisher.
